# Is asymmetric upper trapezius muscle activation during work associated with neck pain? A cross-sectional and longitudinal analysis

**DOI:** 10.1371/journal.pone.0349265

**Published:** 2026-06-12

**Authors:** Markus Koch, Xuelong Fan, Lars Louis Andersen, Markus Due Jacobsen, Mikael Forsman, Gisela Sjøgaard, Karen Søgaard, Christoph Anders, Sanna Grydeland, Lars-Kristian Lunde

**Affiliations:** 1 Research Group for Work Psychology and Physiology, National Institute of Occupational Health, Oslo, Norway; 2 Department of Medical Sciences, Uppsala University, Uppsala, Sweden; 3 The National Research Centre for the Working Environment, Musculoskeletal Disorders and Physical Workload, Copenhagen, Denmark; 4 Institute of Environmental Medicine, Karolinska Institute, Stockholm, Sweden; 5 Division of Ergonomics, School of Engineering Sciences in Chemistry, Biotechnology and Health (CBH), KTH Royal Institute of Technology, Huddinge, Sweden; 6 Department of Sports Science and Clinical Biomechanics, University of Southern Denmark, Odense, Denmark; 7 Department of Clinical Research, University of Southern Denmark, Odense, Denmark; 8 Division of Motor Research, Pathophysiology and Biomechanics, Experimental Trauma Surgery, Germany; University of Study of Bari Aldo Moro, ITALY

## Abstract

**Objectives:**

Previous studies have linked activity in the upper trapezius muscle with neck pain. However, no studies have examined whether asymmetric activation of these muscles during the working day is associated with neck pain. This study aimed to investigate this relationship.

**Methods:**

Seven research institutes provided data on bilateral upper trapezius muscle activity on one working day, along with corresponding questionnaire data on cross-sectional (n = 530) and longitudinal (n = 256) neck pain intensity. The asymmetry, defined as the activity difference between the two upper trapezius muscles, was calculated as an average across the entire workday and within various intensity levels in relation to maximum voluntary isometric contraction (MVIC). Unadjusted and adjusted linear regression analyses were executed to examine the association between asymmetric muscle activation and neck pain intensity.

**Results:**

In cross-sectional analyses, asymmetry in the levels 0–0.05 and 0.05–2%MVIC was significantly positively associated with neck pain intensity in both unadjusted and adjusted analyses. Asymmetry in the levels of 4–6, 6–8 and 8–10%MVIC was significantly negatively associated with neck pain in unadjusted analyses. In longitudinal analyses, significant positive associations were found for asymmetry in level 0–0.05%MVIC and negative associations for asymmetry in levels > 20%MVIC.

**Conclusion:**

While asymmetry in the very low levels of muscle activity may be associated with higher neck pain intensity, asymmetry in the higher levels of muscle activity was negatively associated with neck pain intensity. However, the explained variance of the models was small, and the results should therefore be interpreted with caution. The findings suggest that work conditions facilitating simultaneous relaxation during breaks and balanced activation of both muscles during static activities may be relevant for neck pain prevention, though further research is needed to establish causality.

## Introduction

Neck pain is still one of the most common musculoskeletal disorders and the fourth leading cause of disability worldwide [1], with the highest prevalence rates in the Scandinavian countries [2]. In 2019 the age-standardized rate for prevalence of neck pain was 2,696 per 100,000 population, and in general, the burden of neck pain increases with age and is higher in women than in men [3].

Risk factors for neck pain are multifactorial including individual, psychological and biological factors [4]. Among work related factors, biomechanical exposures as working in awkward or sustained postures [5], work with arms above shoulder height [6], or other body positions [7] may be important precursors of neck pain. Additionally, psychological and social factors [8] at the workplace can contribute to the incidence of neck pain.

The muscle activity in the upper trapezius muscle depicts its physical exposure, when generating forces. With an increasing force use in the muscle, its activity is rising [9]. However, other factors influencing muscle activity can also be psychological, like stress [10], mental or emotional load of working tasks [11], general tension, and the organization of work and breaks [12,13].

Previous studies have examined the association between upper trapezius muscle activity and neck pain during the workday. These studies have focused on mean muscle activity [14–16], muscle rest [17,18], or sustained muscle activity [13,19], mainly in individual muscles. However, to our knowledge, no studies have examined asymmetric upper trapezius muscle activity throughout the workday, especially performed at the workplace itself.

Numerous studies have examined the co-activation of agonist and antagonist muscles. Research on co-activation is the opposite approach to studying asymmetry in muscle activation. Co-activation refers to the simultaneous contraction of agonist–antagonist muscles, where muscles on both sides of the same joint are active together. Asymmetry, by contrast, captures the degree to which one side’s activation (right vs left) differs from the other at a given moment. The results of co-activation studies are mixed, and a few studies suggest that asymmetry may be a concern for the neck. As for example in a laboratory setting, De Almeida and co-authors were observing co-activation of the upper trapezius muscles of dental students throughout their initial semester of clinical training. While performing a cranio-cervical flexion, students with neck pain showed increased co-activation in the upper trapezius muscles compared to their healthy students [20].

A previous study measuring the asymmetry in muscle activation of various back muscles during contraction in twisted postures, found an increasing asymmetry with increasing angle of rotation of the trunk [21]. The study did not include the upper trapezius muscle, however, the authors concluded that an asymmetric muscle activation will propose various failure mechanisms in the spine, including a one-sided strain on the vertebrae, as a possible cause for low back pain. Comparable mechanisms can be expected for the cervical spine with the corresponding muscles. Findings contradicting this theory were found by Lindstrøm and co-authors [22]. Their study examined the co-activation of sternocleidomastoid (SCM) and splenius capitis (SC) muscles in various movements of the neck. Compared to healthy control group, patients with neck pain showed higher coactivation in the mentioned muscles. Additionally, the increased coactivation of the splenius capitis muscle was found to be associated with lower neck strength and higher levels of pain [22].

In contrast to laboratory studies, a full working day consists of various movements, strength requirements, and static working positions depending on the occupational group or specific work tasks. The upper trapezius muscles must work both asymmetrically and co-actively to stabilize the cervical spine or enable movement. It is unclear whether the activation of the upper trapezius muscles in an asymmetrical or co-active manner during the working day affects the occurrence of neck pain. Understanding this relationship could inform targeted workplace interventions and ergonomic strategies to prevent neck pain by optimizing bilateral muscle coordination patterns during occupational tasks.

This study examined the asymmetry in the upper trapezius muscle activity throughout the working day and explored its cross-sectional and longitudinal associations with neck pain intensity. We hypothesized that an asymmetry in activation of the upper trapezius muscle is associated with neck pain intensity.

## Methods

### Dataset

Activity of the upper trapezius muscles recorded by surface electromyographic (EMG) of one working day and questionnaire data containing information about neck pain intensity and individual factors were provided from seven Scandinavian research institutes for 748 participants [16,19,23–37]. The protocol of this study provides a comprehensive overview of the individual measurement protocols employed in the included studies, along with a detailed exposition of the methodology employed to address existing differences [38]. The data contained in each individual study was collected between 01.11.2021 and 30.06.2022 in anonymized form. The different datasets were merged into a common format, and questions on neck pain were harmonized. Written consent was obtained from all subjects involved in the included studies.

### Study population

For 530 participants bilateral data of trapezius muscle activity was available (284 men, 246 women). The study population included workers in 26 different professions (Table 1). The average age, weight, height and BMI of the participants were 39.2 years (SD 12.5 years), 75.4 kg (SD 15.1 kg), 173.7 cm (SD 9.6 cm), and 24.9 kg/m2 (SD 3.6 kg/m2) (see Table 2).

**Table 1:** Descriptive statistics of the study population.

		Cross-sectional analyses		Longitudinal analyses	
		n	Percent	n	Percent
**Gender**	Male	284	53.6	142	55.5
	Female	246	46.4	114	44.5
**Smoking**	No	222	63.4	142	69.3
	Yes	128	36.6	63	30.7
	Missing	180		51	
**Dominant hand**	Right	273	82.5	150	75.4
	Left	58	17.5	49	24.6
	Missing	199		57	
**Profession**	Assembly worker	11	2.1		
	Assistant worker	2	0.4	1	0.4
	Bricklayer	22	4.2	1	0.4
	Carpenter	17	3.2	10	3.9
	Cleaner	2	0.4	2	0.8
	Concrete worker	37	7.0	5	2.0
	Cook or kitchen helper	8	1.5	2	0.8
	Electrician	16	3.0	11	4.3
	Engineer	3	0.6	2	0.8
	Foreman	5	0.9	3	1.2
	Hairdresser	35	6.6	29	11.3
	Harvester/ Driver	84	15.8	83	32.4
	Health care personnel	70	13.2	33	12.9
	Helicopter pilot	18	3.4		
	Meat cutter	8	1.5		
	Office worker/ Secretary	93	17.5	43	16.8
	Project manager/ leader	8	1.5	7	2.7
	Retail personal	27	5.1	11	4.3
	Rubber mixing	8	1.5		
	Student	5	0.9	4	1.6
	Surgeon	21	4.0		
	Warehouse worker	3	0.6		
	Windscreen inspection	10	1.9		
	Working with various tasks	6	1.1	3	1.2
	Other occupations	11	2.1	6	2.3
**Total**	**Total**	**530**	**100.0**	**256**	**100.0**

**Table 2:** Descriptive statistics of the dataset.

	Cross-sectional analyses			Longitudinal analyses		
	N	Mean	SD	N	Mean	SD
***Individual’s constitution***						
Age [years]	517	39.2	12.5	252	38.2	12.6
Weight [kg]	489	75.4	15.1	249	75.8	15.2
Height [cm]	496	173.7	9.6	255	174.0	9.6
BMI [kg/m^2^]	491	24.9	3.6	249	24.9	3.6
***Neck pain intensity***						
Cross-sectional [VAS]	530	1.7	2.3			
Longitudinal [VAS]				256	1.9	2.0
***Asymmetry in muscle activation***						
Average highest side activity [% MVIC]	530	7.3	3.9	256	7.2	3.4
***Average of side independent asymmetry [%]***						
***Levels related to MVIC:***						
0–0.05%	530	45.1	12.8	256	44.2	13.8
0.05–2%	530	52.9	11.1	256	53.3	11.5
2 - 4%	530	53.8	9.8	256	54.2	10.0
4 - 6%	530	52.3	10.0	256	52.7	10.6
6 - 8%	530	51.9	10.1	256	52.4	10.8
8 - 10%	530	52.2	10.1	256	52.7	10.9
10 - 20%	530	54.3	10.2	256	55.1	11.3
20 - 50%	530	59.5	10.5	256	60.9	11.7
50 - 100%	530	60.6	18.8	254	64.4	15.4
**Full range *(0–100%)***	530	51.6	7.8	256	52.0	8.7
***Average of side dependent asymmetry [%]***						
***Levels related to MVIC:***						
0–0.05%	530	18.2	13.7	256	18.0	12.9
0.05–2%	530	19.6	15.3	256	19.8	15.6
2 - 4%	530	19.7	16.0	256	19.7	16.4
4 - 6%	530	20.1	16.1	256	20.3	16.8
6 - 8%	530	20.5	16.2	256	21.0	16.7
8 - 10%	530	21.3	16.1	256	21.8	16.8
10 - 20%	530	22.8	17.1	256	23.9	18.0
20 - 50%	530	27.7	19.4	256	29.2	20.9
50 - 100%	530	37.1	25.4	254	39.3	26.0
**Full range *(0–100%)***	530	18.1	14.1	256	18.1	14.7

Cross-sectional analyses were conducted on all 530 participants, while longitudinal analyses were only conducted for those with longitudinal data on neck pain intensity (n = 256).

### Objective measures – EMG

Every study followed recommendations and standards of EMG measurements, including sensor placement and data processing [39,40]. A detailed overview of the positions for sensor placement, measurement frequency and positions for examining a reference measurement with maximum voluntary isometric contraction (MVIC) is given in the protocol paper of this study [38]. During pooling, all EMG recordings were transformed into a standard root mean square format (RMS) of 8 Hz, normalized to MVIC and quality controlled. Each individual EMG recording was corrected for noise by subtracting the noise level (minimum of the average of a moving average over 19 samples) from all samples [41,42].

### Asymmetry in muscle activity

In this study, asymmetry in bilateral muscle activation is defined as the difference between the activity of both upper trapezius muscles. To calculate this asymmetry, e.g., in normalized muscle activity or conditioning, various equations have been used before [43]. We defined the side-independent asymmetry of a subject as the average proportion of the absolute difference between the left and right upper trapezius muscle activity relative to the highest activity of both upper trapezius muscles, computed across the entire working day. It was calculated as:


$$\begin{array}{c} \overset{\left(side\;independent\right)}{\underset{k}{Asymmetry\;}}=\frac{\text{1}}{T}\sum_{i=\text{1}}^{T}\left(\frac{\left|{Right}_{i}-\;{Left}_{i}\;\right|}{\text{max}({Right}_{i},\;{Left}_{i})}\right)\times \text{1}\text{0}\text{0}\% \end{array}$$ (1)

where $\overset{\left(side\;independent\right)}{\underset{k}{Asymmetry}}$ is the averaged side-independent asymmetry for subject k; ${Right}_{i}$ and ${Left}_{i}$ denote the muscle activity of the right and left trapezius at time i; and T is the duration (number of time points) of the recorded working time.

In this definition of asymmetry, a result of 0% indicates perfectly symmetrical activity, while values approaching 100% reflect increasingly unbalanced activity, with one side exhibiting much higher activation than the other. This definition does not take the side of the asymmetry into account.

Similarly, to specify the side of the asymmetry, we defined the side-dependent asymmetry of a subject as the average proportion of the difference between the left and right upper trapezius muscle activity relative to the highest activity of both muscles, computed across the entire working day. It was calculated as:


$$\begin{array}{c} \overset{\left(side\;dependent\right)}{\underset{k}{Asymmetry\;}}=\frac{\text{1}}{T}\sum_{i=\text{1}}^{T}\left(\frac{{Right}_{i}-\;{Left}_{i}\;}{\text{max}({Right}_{i},\;{Left}_{i})}\right)\times \text{1}\text{0}\text{0}\% \end{array}$$ (2)

where *Asymmetry*_*k*_^*(side dependent)*^ is the averaged side-dependent asymmetry for subject k; ${Right}_{i}$ and ${Left}_{i}$ denote the muscle activity of the right and left trapezius at time i; and T is the duration (number of time points) of the recorded working time.

**Fig 1:**
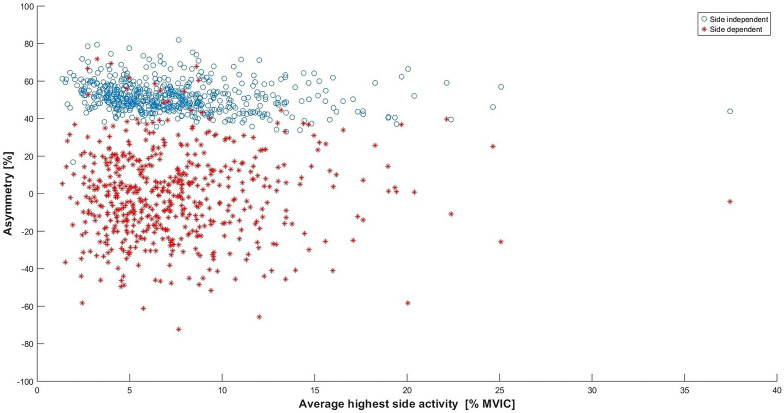
Asymmetry in activity of upper trapezius muscles. Mean asymmetry related to the average highest side activity [% MVIC] of the individual EMG recordings (n = 530); Blue: side independent asymmetry; Red: side dependent asymmetry; The side-dependent mean values of asymmetry are shown with a sign. Positive values mean that muscle activity in the left upper trapezius was, on average, greater than in the right. Negative values indicate that the activity in the right upper trapezius muscle was higher on average. In the statistical analyses, the mean values of the side-dependent asymmetry were included as absolute values.

In addition, to determine the overall muscle activity level on which the asymmetry is based, the average highest side activity was calculated for each participant over the entire recorded working time as follows:


$$\begin{array}{c} {Average\;highest\;side\;activity\;}_{k}=\frac{\text{1}}{T}\sum_{i=\text{1}}^{T}\text{max}({Right}_{i},\;{Left}_{i})\times \text{1}\text{0}\text{0}\% \end{array}$$ (3)

where *Average highest side activity*_*k*_ represents the mean of the highest activity of both upper trapezius muscles for subject k; ${Right}_{i}$ and ${Left}_{i}$ denote the muscle activity of the right and left trapezius at time i; and T is the total number of time points during the recorded working time. An overview of the calculated means of asymmetry in upper trapezius muscles activity and their overall muscle activity level in relation to MVIC is given in Fig 1.

Additionally, for various levels of the upper trapezius muscle activity in relation to MVIC (0% – 0.5%, 0.5% − 2%, 2% − 4%, 4% − 6%, 6% − 8%, 10% – 20%, 20% – 50% and 50% – 100%) both the side independent and the side dependent mean asymmetry was calculated. The maximum value of bilateral muscle activity for each sample was used to assign the asymmetry into these ranges. This additional classification was made because the amplitude of muscle activation corresponds to the force generated by the muscle, which must be applied during various types of work task, for example. A muscle activation < 0.5%MVIC represents muscular rest, when the muscle is assumed to be recovering [44]. Previous studies have defined levels from 0.5–7.0%MVIC as medium activity, and > 7.0%MVIC as high activity [45,46]. Due to the wide range of professions included, we choose a more detailed classification.

### Subjective measures – neck pain

Neck pain intensity was measured by questionnaire, and questions varied both for temporal occurrence and in the measuring scale between the various institutes/conducted studies. For cross-sectional neck pain, measured after the EMG-recording, the included time periods for occurrence of neck pain were “pain after the working day”, “pain for the past seven days”, “pain for the past 4 weeks” and “pain for the past 12 months”. Pain was rated on a visual analog scale (VAS), on a 3-point scale (no pain, slightly bothered, quite bothered) and a 4-point scale (no pain, slight pain, substantial pain, very bothered). The values of the categorial scales were transformed to values of the VAS. A value of zero was retained to represent “no pain.” In the case of the 4-point scale, the values were replaced with zero, three, five, and seven, respectively. Similarly, the 3-point scale was substituted with the values zero, three, and six. The VAS was selected as the primary scale for the pooled dataset with a view to averting any further deterioration in the quality of the pain measurements. The allocation of individual categorical responses to corresponding numerical values on the VAS was determined by consent among all co-authors based on a “best fit”.

Longitudinal occurrence of neck pain was available for 256 participants and was recorded after six, twelve and 24 months. Neck pain intensity was rated on a VAS and a 4-point scale (no pain, slight pain, substantial pain, very bothered) and the values of the 4-point scale were transformed into VAS values as described above.

### Statistics

Associations between individual factors and neck pain were examined with linear regression analyses. Significant individual factors associated with neck pain (see S1 Table) were included as confounders in the various models when examining the association between the asymmetry in upper trapezius muscles activation and neck pain.

Associations between parameters describing the asymmetrical activity of the upper trapezius muscles (both for side independent and side dependent asymmetry: mean, center, mean in various levels of MVIC) and neck pain intensity were examined with linear regressions both unadjusted and adjusted. Confounders were selected based on their biological plausibility as risk factors for neck pain. For the cross-sectional analyses, Model 1 included the unadjusted associations between asymmetry-variables and neck pain intensity. Model 2 was adjusted for sex, as women consistently show higher neck pain prevalence and different muscle activation patterns compared to men [3]. Model 3 was additionally adjusted for height, which influences biomechanical loading and muscle activation requirements during work tasks [47]. Model 4 additionally included smoking, given its association with musculoskeletal pain [48].

In the longitudinal analyses Model 1 included the unadjusted associations between asymmetry-variables and neck pain intensity, in Model 2 the associations were adjusted for sex and in Model 3 the associations were adjusted for sex, height.

The results of the regression analyses are presented as β coefficients (change in VAS pain per one-percentage-point increase in asymmetry or higher activity-side load), alongside adjusted R² and p-values.

### Ethics

The consent for the pooling, storing and analysis of the various anonymized datasets was given by the Norwegian Centre for Research Data. Because ethical approval was already obtained in the original studies of the pooled data, no need for further ethical approval was necessary (Regional Committee for Medical Research Ethics (Act on Medical and Health Research §§2. section 1.4.d. Application title: “Muskelaktivering og pauser under arbeid og nakkesmerter”; application number: 275929; REK sør-øst B. Gullhaugveien 1–3. 0484 Oslo).

## Results

On average participants rated neck pain intensity 1.7 (SD 2.3) and 1.9 (SD 2.0) in cross-sectional in longitudinal measures (see Table 2).

Across all participants, the average asymmetry in activation of both upper trapezius muscles during the working day was 51.6% (SD 7.8%) and the average highest side activity was 7.3% (SD 3.9%. For the various levels of muscle activity in relation to their respective MVIC the mean asymmetry during the working day varied from 45.1% (SD 12.8%) to 60.6% (SD 18,8%) (see Table 2 and Fig 1).

### Cross-sectional analyses of asymmetry of muscle activation and neck pain intensity

In cross-sectional analyses, the average highest side activity in relation to MVIC was negatively related to neck pain intensity both in Model 1 (β −0.097, R^2^ 0.009, p < 0.05) and Model 2 (β −0.097, R^2^ 0.053, p < 0.05). With the average highest side activity in lower amplitudes related to the MVIC, participants showed a higher pain intensity (see Table 3).

**Table 3:** Regression analysis between asymmetric activation of upper trapezius muscles and cross-sectional neck-pain (n = 530).

	Model 1			Model 2			Model 3			Model 4		
	β	R2 (adj.)	p	β	R2 (adj.)	p	β	R2 (adj.)	p	β	R2 (adj.)	p
Average highest side activity	**−0.097**	**0.009**	**< 0.05**	**−0.097**	**0.053**	**< 0.05**	−0.083	0.047		−0.005	0.025	
**Side independent asymmetry**												
***Levels related to MVIC:***												
0–0.05%	**0.181**	**0.033**	**< 0.001**	**0.181**	**0.088**	**< 0.001**	**0.219**	**0.085**	**< 0.001**	0.087	0.031	
0.05–2%	**0.162**	**0.026**	**< 0.001**	**0.162**	**0.073**	**< 0.001**	**0.165**	**0.067**	**< 0.001**	−0.034	0.026	
2 - 4%	0.027	0.001		0.027	0.045		0.033	0.041		−0.017	0.026	
4 - 6%	−0.072	0.005		−0.072	0.046		−0.039	0.042		−0.016	0.026	
6 - 8%	**−0.11**	**0.012**	**< 0.05**	−0.11	0.048		−0.054	0.043		−0.004	0.025	
8 - 10%	**−0.116**	**0.014**	**< 0.01**	−0.116	0.047		−0.046	0.042		−0.003	0.025	
10 - 20%	**−0.086**	**0.007**	**< 0.05**	−0.086	0.045		−0.005	0.04		0.025	0.026	
20 - 50%	−0.063	0.004		−0.063	0.044		0.014	0.04		0.069	0.03	
50 - 100%	−0.008	0.000		−0.008	0.043		0.037	0.04		0.1	0.035	
**Full range (0–100%)**	−0.011	0.000		−0.011	0.045		0.042	0.042		−0.013	0.026	
**Side dependent asymmetry**												
***Levels related to MVIC:***												
0–0.05%	0.022	0.000		0.022	0.045		0.049	0.041		−0.004	0.025	
0.05–2%	0.025	0.001		0.025	0.046		0.052	0.043		0.03	0.026	
2 - 4%	−0.049	0.002		−0.049	0.045		−0.036	0.042		−0.038	0.027	
4 - 6%	**−0.097**	**0.009**	**< 0.05**	−0.097	0.049		−0.081	0.047		−0.08	0.032	
6 - 8%	**−0.103**	**0.011**	**< 0.05**	−0.103	0.049		−0.074	0.046		−0.085	0.032	
8 - 10%	**−0.095**	**0.009**	**< 0.05**	−0.095	0.048		−0.06	0.044		−0.095	0.034	
10 - 20%	−0.074	0.006		−0.074	0.046		−0.04	0.042		−0.073	0.03	
20 - 50%	−0.048	0.002		−0.048	0.045		−0.022	0.041		0.021	0.026	
50 - 100%	−0.006	0.000		−0.006	0.041		0.005	0.036		0.015	0.025	
**Full range (0–100%)**	−0.056	0.003		−0.056	0.045		−0.027	0.041		−0.042	0.027	

Model 1: Association between asymmetry in upper trapezius muscles activation and cross-sectional neck pain

Model 2: Model 1 adjusted for sex

Model 3: Model 1 adjusted for sex, height

Model 4: Model 1 adjusted for sex, height, smoking

When examining the asymmetry without an assignment to one side of the body, asymmetry in the levels from 0 to 0.5%MVIC and 0.05 to 2.0%MVIC was significantly positively associated with neck pain intensity in Model 1 (0 to 0.5% MVIC: β 0.181, R^2^ 0.033, p < 0.001; 0.05 to 2.0%MVIC: β 0.162, R^2^ 0.026, p < 0.001), Model 2 (0 to 0.5% MVIC: β 0.181, R^2^ 0.088, p < 0.001; 0.05 to 2.0%MVIC: β 0.162, R^2^ 0.073, p < 0.001) and Model 3 (0 to 0.5%MVIC: β 0.219, R^2^ 0.085, p < 0.001; 0.05 to 2.0%MVIC: β 0.165, R^2^ 0.067, p < 0.001). In the levels of 6–8%MVIC (β −0.11, R^2^ 0.012, p < 0.05), 8–10% MVIC (β −0.116, R^2^ 0.014, p < 0.01), and 10–20%MVIC (β −0.086, R^2^ 0.007, p < 0.05), asymmetry was significantly negatively associated with neck pain intensity in Model 1. The significant associations that were identified were found to be of a small magnitude (β < 0.181), as was the variance in neck pain intensity that was explained (R² < 0.088).

Taking the assignment to body side of the asymmetry of the muscle activity into account, significant negative associations were found between the asymmetry of the muscle activation in the levels of 4–6%MVIC (β −0.097, R^2^ 0.009, p < 0.05), 6–8%MVIC (β −0.103, R^2^ 0.011, p < 0.05), and 8–10%MVIC (β −0.095, R^2^ 0.009, p < 0.05) in unadjusted analyses. The significant associations that were identified were found to be of a small magnitude (β < 0.103) and explained a low variance in neck pain intensity (R² < 0.011).

No significant results were found in the cross-sectional analyses of Model 4.

### Longitudinal analyses of asymmetry of muscle activation and neck pain intensity

Compared to the cross-sectional analyses, the longitudinal analyses yielded fewer and weaker significant associations overall. In longitudinal analyses that did not account for laterality (i.e., side-independent asymmetry), we found only one significant negative association between the asymmetry of muscle activity in the range of 20–50%MVIC (β −0.150, R^2^ 0.023, p < 0.05) and neck pain intensity in unadjusted analyses (see Table 4).

**Table 4:** Regression analysis between asymmetric activation of upper trapezius muscles and longitudinal neck-pain (n = 256).

	Model 1			Model 2			Model 3		
	β	R2 (adj.)	p	β	R2 (adj.)	p	β	R2 (adj.)	p
Average highest side activity	0.019	0.000		0.001	0.016		0.002	0.038	
**Side independent asymmetry**									
***Levels related to MVIC:***									
0–0.05%	0.022	0.000		0.078	0.022		0.062	0.041	
0.05–2%	−0.009	0.000		0.026	0.017		0.032	0.039	
2–4%	−0.009	0.000		0.004	0.016		0.027	0.039	
4–6%	−0.048	0.002		−0.028	0.017		−0.006	0.038	
6–8%	−0.082	0.007		−0.050	0.018		−0.030	0.039	
8–10%	−0.099	0.010		−0.062	0.020		−0.046	0.040	
10–20%	−0.116	0.013		−0.077	0.021		−0.072	0.042	
20–50%	**−0.150**	**0.023**	**<0.05**	−0.120	0.029		−0.114	0.049	
50–100%	−0.106	0.011		−0.071	0.019		−0.062	0.040	
**Full range (0–100%)**	−0.072	0.005		−0.037	0.017		−0.029	0.039	
**Side dependent asymmetry**									
***Levels related to MVIC:***									
0–0.05%	**0.121**	**0.015**		**0.144**	**0.037**	**<0.05**	**0.127**	**0.054**	**<0.05**
0.05–2%	0.077	0.006		0.098	0.026		0.094	0.046	
2 - 4%	0.003	0.000		0.024	0.017		0.035	0.039	
4 - 6%	−0.029	0.001		−0.002	0.016		0.012	0.038	
6 - 8%	−0.037	0.001		−0.007	0.016		−0.002	0.038	
8 - 10%	−0.063	0.004		−0.035	0.017		−0.037	0.039	
10 - 20%	−0.088	0.008		−0.068	0.021		−0.073	0.043	
20 - 50%	**−0.142**	**0.020**	**<0.05**	**−0.125**	**0.031**	**<0.05**	−0.119	0.052	
50 - 100%	**−0.190**	**0.036**	**<0.01**	**−0.169**	**0.041**	**<0.01**	**−0.171**	**0.064**	**<0.01**
**Full range (0–100%)**	−0.023	0.001		0.003	0.016		0.011	0.038	

Model 1: Association between asymmetry in upper trapezius muscles activation and longitudinal neck pain (unadjusted)

Model 2: Model 1 adjusted for sex

Model 3: Model 1 adjusted for sex and height

When accounting for laterality (side-dependent), significant negative associations between the asymmetry of muscle activity in the levels of 4–6%MVIC (β −0.097, R^2^ 0.009, p < 0.05), 6–8%MVIC (β −0.103, R^2^ 0.011, p < 0.05), and 8–10%MVIC (β −0.095, R^2^ 0.009, p < 0.05) and neck pain were found, when not adjusting for possible confounders.

## Discussion

This study examined the association between asymmetric activation of the upper trapezius muscles and neck pain intensity in short and long term.

### Pain intensity and study population characteristics

The values of the neck pain intensity measured in our study were 1.7 (SD 2.3) and 1.9 (SD 2.0) on the VAS for cross-sectional and longitudinal measures, respectively. These values are lower compared to those of population-based studies in Denmark [49] or Norway [50]. This lower pain prevalence may have attenuated the strength of associations observed between upper trapezius muscle asymmetry and neck pain intensity.

### Asymmetry patterns and occupational context

The calculated average values of the asymmetry during the working day were 51.6% for the cross-sectional and 52.0% for the longitudinal analyses, with an average highest side activity of 7.3%MVIC and 7.2%MVIC, respectively. The values for the average highest side activity reflect the expected distribution of muscle activity during typical work tasks, where most activities occur in lower muscle activation levels corresponding to postural maintenance and light manual tasks [36]. Since one can assume that most of the working day is spent with lower muscle activity levels, the average highest side activity is comprehensible. An earlier study examined the activation of the upper trapezius muscle during a workday in a wide range of occupational groups [36]. On average, the total population was found to have a static level (10th percentile) and a peak level (90th percentile) of 1.5%MVIC (SD 1.0%MVIC) and 14%MVIC (5%MVIC), respectively. The average highest side of muscle activity, which we determined, lies within this range. Therefore, concerning this asymmetry, our study can be considered representative of the general working population.

The values for asymmetry in the various levels related to MVIC ranged from 44.2% to 64.4%, with increasing values for higher muscle activation levels. This trend is physiologically reasonable, as higher-intensity tasks often require unilateral movements or force generation, naturally leading to greater side-to-side differences in muscle activation.

### Cross-sectional association between asymmetric muscle activation and neck pain intensity

Our cross-sectional analyses revealed a differential relationship between asymmetry and neck pain intensity across different muscle activation levels. Asymmetry in very low activation levels (0–0.05 and 0.05–2%MVIC) was positively associated with neck pain intensity, while asymmetry in moderate levels (6–10%MVIC) showed protective effects. This pattern indicates that the relationship between asymmetric muscle activation and pain is not linear but rather depends on the functional context of muscle activation.

The detrimental effect of asymmetry during near-rest conditions (0–2%MVIC) may reflect impaired bilateral motor control during postural maintenance and recovery periods [44]. During these low-level activities, symmetric activation is crucial for optimal load distribution across cervical structures [51]. Asymmetric patterns during rest periods may indicate underlying neuromuscular dysfunction, compensation strategies, or inadequate recovery, potentially contributing to tissue stress and pain development. However, it should be noted that the observed association may not be attributable to asymmetry itself, but rather to sustained low-level muscle activity or insufficient muscular rest, both of which have previously been linked to neck pain [13,17]. The asymmetry measure at these very low activation levels may therefore partly capture these established risk factors rather than reflecting an independent effect of bilateral imbalance.

Conversely, the protective effect of asymmetry in moderate to higher activation levels likely reflects appropriate task-specific motor control strategies, where asymmetric activation serves functional purposes such as tool manipulation, load handling, or directional movements. In these contexts, one side naturally dominates based on functional demands and task requirements rather than representing pathological compensation patterns. This suggests that some degree of asymmetry during active work tasks may be beneficial and represents normal adaptive motor behavior rather than dysfunction. However, the counterintuitive finding that higher asymmetry appears protective warrants consideration of potential residual confounding factors that our models may not have fully captured, such as work technique quality, individual motor control capabilities, or unmeasured occupational exposures. These interpretations remain speculative and should be confirmed in future experimental studies.

An important consideration is that the observed associations may reflect reverse causality – existing neck pain could lead individuals to favor the non-painful side, creating asymmetric activation patterns rather than asymmetry in muscle activation causing pain. Our cross-sectional design cannot distinguish between these causal directions.

Including smoking in the regression analyses (Model 4) resulted in none of the associations remaining significant. Particularly for cross-sectional associations, smoking appears to have a large effect on the occurrence of neck pain. When examining possible confounders for neck pain (see S1 Table), we found a significant negative association in cross-sectional analyses. This finding contrasts with other studies, which have generally found higher levels of pain associated with smoking [52]. One possible explanation for the negative associations between smoking and neck pain intensity might be the opportunity to rest during smoking breaks. It is also conceivable that the pain is alleviated by increased social interaction during these breaks [4]. Due to the small number of smokers (n = 128) in our sample, this association was not investigated further.

### Longitudinal association between asymmetric muscle activation and neck pain intensity

Longitudinal analyses revealed fewer significant associations for side-independent asymmetry. Only asymmetry at the 20–50%MVIC level was negatively associated with neck pain intensity. Taking side-dependency of the asymmetry into account, significant negative associations were found between asymmetry at muscle activity levels greater than 20%MVIC and neck pain intensity. Conversely, positive associations between asymmetry during muscular rest and neck pain intensity, when considering side dependency, could lead to a constant uneven vertebral bracing or vertebral positioning, which may increase pain in the long term [51]. Due to the lack of information about the position of the spine and the additional load it carries, this can only be hypothesized. De Almeida and co-authors found an increased co-activation in the upper trapezius muscles while performing a cranio-cervical flexion in students with neck pain compared to their healthy students [20]. Both their and our results imply that the ability to generate asymmetric muscle activation during high-intensity activities may protect against future neck pain by reflecting better neuromuscular function and adaptability. However, this hypothesis is contradicted by the results of an earlier study. Szeto et al. conducted a study to examine the median activity of the left and right trapezius muscles during a one-hour computer typing task in a sample of office workers [53]. Their study revealed a greater difference in median activity between the left and right muscles in participants who reported neck discomfort during the previous 12 months. Given that median values for upper trapezius muscle activity ranged from approximately 11–21%MVIC, it can be deduced that, in the context of the typing task, the asymmetry, according to the formula we used, would have been higher for the case-group. Since their task included no greater body movements at all, it can only be speculated, why the participants in their case group developed an asymmetric pattern over time. Unfortunately, their study was not carried out in a longitudinal design, and many other external factors may have contributed to the development of an altered pattern.

The small number of significant findings in general, and specifically their small magnitude and the low values of explained variance in neck pain intensity in the analyses may reflect the complex, multifactorial nature of neck pain development over time, where acute muscle activation patterns represent only one of many risk factors [8,11].

### Strengths and limitations

When analyzing the association between upper trapezius muscle activity and neck pain, examining the asymmetry in upper trapezius muscle activation is a strength. This approach is the opposite of analyzing the co-activation or co-contraction of several muscles, as has been done in previous studies. In general, one might question whether this new approach simply yields reversed results or associations. To have this discussion, studies focusing on co-activation must have used formulas like the one we used for asymmetric muscle activation. Despite the number of studies on co-contraction or co-activation, there does not seem to be a gold standard for calculating co-activation or co-contraction. For example, a recent review identified four different formulas used in the included studies [54]. Various formulas can also be found for calculating asymmetry [43], which makes it difficult to compare different studies on co-activation and asymmetry. For supplementary analyses, we calculated the co-activation of the upper trapezius muscles during the working day according to the formula used by Hammond et al. [55]. Analyses of co-activation and neck pain intensity revealed associations that were the opposite of those observed for asymmetry. These associations were mostly significant at similar levels of muscle activity in both the cross-sectional (0–0.05, 0.05–2, 6–8, and 8–10%MVIC) and longitudinal (20–50%MVIC) analyses (see S2 and S3 Table).

A major strength of this study is the number of participants included in both the cross-sectional and longitudinal analyses. By combining individual data sets, it was possible to create such a database containing muscle activity measurements over an entire working day with corresponding measurements of neck pain intensity. The data set contains 26 occupational groups, which are characterized by broad ergonomic and psychological stresses, and can thus be seen as a reflection of a general working population.

A potential limitation of this study is that the questions regarding neck pain intensity differed across the included studies. The temporal parameters of the occurrence of neck pain exhibited variability, as did the scales utilized to assess neck pain intensity. As authors, we endeavored to achieve the most optimal consensus in pooling the questionnaire data. To maintain the quality of the pain intensity scale while maximizing the number of participants, the 3-point and 4-point scales were converted to VAS. This transformation may have biased the analysis of the association between the asymmetry of muscle activity and pain intensity measures.

It can also be discussed whether the quality of the EMG measurements was of the same high quality in all included studies. In essence, the veracity of this assertion cannot be ascertained through a retrospective examination. However, the quality criteria for the EMG measurements were met in all studies, and the parameters for the measurements were documented in the individual studies. The extent to which different body positions for the reference contention (MVIC) influence the subsequent muscle activity values remains a subject of speculation. However, given that no direct comparison is being made between the measured values from these individual studies, this potential discrepancy may be disregarded. In the present study, an investigation was conducted to compare the muscle activity of the two upper trapezius muscles. Given that this data originates from a corresponding measurement, a high degree of accuracy can be expected, particularly since all data were visually reviewed by us for quality before analysis.

During the process of quality control, the noise present in the signal of the activation of the upper trapezius muscles was systematically removed from all channels. Furthermore, both signals (left and right muscle activity) were measured in individual sub-studies using a single measuring device. Given that the noise level was typically less than 1% MVIC, the impact of an incorrect calculation of the noise level on the asymmetry values can be considered negligible. The repercussions of such an error would be most significant in areas exhibiting minimal muscle activation. However, given the tendency of asymmetry values to increase from areas of low muscle activation to those of high muscle activation, it can be assumed that asymmetry values can be regarded as reliable.

The asymmetry of upper trapezius muscle activation was calculated for each sample using an RMS window of 8 Hz for the EMG measurements, which is debatable. One could also calculate a moving average with various lengths (e.g., one or two seconds) to see how it influences the amount of asymmetry in muscle activation and the association between asymmetric upper trapezius muscle activation and neck pain intensity.

Despite the identification of several substantial associations, the corresponding values for the explained variances in neck pain intensity were found of a small magnitude. Consequently, the influence of a potential asymmetric activation in the upper trapezius muscle on neck pain intensity is estimated to be low. In contrast to laboratory studies, in which specific exposures can be tested, the physical, psychological, or social stresses present in the workplace are wide. The present study was not equipped to provide precise information regarding these exposures. Nevertheless, this wide range of potential risk factors may have contributed to a lower level of variance. While this may be considered a disadvantage, it could also imply that the significant associations identified can be generalized.

### Concluding remarks

By integrating data sets from multiple previous studies, this study examined the association between upper trapezius muscle activity and neck pain intensity, with a focus on the asymmetric activation of these muscles. In contrast to the findings of preceding studies, a novel approach that has not yet been considered was investigated. The results of this study point towards, that asymmetry in the very low levels of muscle activity (muscular rest and static activities) may increase neck pain intensity, while asymmetry in the higher levels of muscle activity appears to be protective against neck pain. In general, the variance explained of the models was small, and the results should therefore be interpreted with caution. The findings suggest that work conditions facilitating simultaneous relaxation during breaks and balanced activation of both muscles during static activities may be relevant for the prevention of neck pain, though further research is needed to establish causality and, if this is the case, to determine what constitutes a ‘normal’ or ‘safe’ level of asymmetrical work.

## Supporting information

S1 Table Regression analyses between individual factors and neck pain. (DOCX)

S2 Table Regression analysis between co-activation of upper trapezius muscles and cross-sectional neck-pain (n = 530). Model 1: Association between asymmetry in upper trapezius muscles activation and cross-sectional neck pain; Model 2: Model 1 adjusted for sex; Model 3: Model 1 adjusted for sex, height; Model 4: Model 1 adjusted for sex, height, smoking.
(DOCX)

S3 Table Regression analysis between co-activation of upper trapezius muscles and longitudinal neck-pain (n = 256). Model 1: Association between asymmetry in upper trapezius muscles activation and longitudinal neck pain (unadjusted), Model 2: Model 1 adjusted for sex; Model 3: Model 1 adjusted for sex and height.
(DOCX)

S1 Dataset Data file for analysis. (XLSX)
