# Supplementary material for: Is asymmetric upper trapezius muscle activation during work associated with neck pain? A cross-sectional and longitudinal analysis
Source: PLoS One. 2026 Jun 12;21(6):e0349265. doi: 10.1371/journal.pone.0349265 (PMC13262944; doi:10.1371/journal.pone.0349265)
Supplement: S1 Table — (DOCX) [file pone.0349265.s001.docx]

S1 Table. Regression analyses between individual factors and neck pain

|  | **Cross-sectional**  **neck pain** | | | **Longitudinal**  **neck pain** | | |
| --- | --- | --- | --- | --- | --- | --- |
|  | **Beta** | **R2 (adj.)** | **p** | **Beta** | **R2 (adj.)** | **p** |
| Sex | **0.208** | **0.043** | **< 0.001** | **0.127** | **0.012** | **< 0.05** |
| Age | 0.060 | 0.002 |  | **0.149** | **0.018** | **< 0.05** |
| Weight | -0.053 | 0.000 |  | -0.020 | -0.004 |  |
| Height | **-0.110** | **0.009** | **< 0.05** | -0.092 | 0.005 |  |
| BMI | -0.029 | -0.001 |  | 0.033 | -0.002 |  |
| Smoking | **-0.124** | **0.014** | **< 0.05** | 0.064 | -0.001 |  |
