# Supplementary material for: Is asymmetric upper trapezius muscle activation during work associated with neck pain? A cross-sectional and longitudinal analysis
Source: PLoS One. 2026 Jun 12;21(6):e0349265. doi: 10.1371/journal.pone.0349265 (PMC13262944; doi:10.1371/journal.pone.0349265)
Supplement: S2 Table — Model 1: Association between asymmetry in upper trapezius muscles activation and cross-sectional neck pain; Model 2: Model 1 adjusted for sex; Model 3: Model 1 adjusted for sex, height; Model 4: Model 1 adjusted for sex, height, smoking. (DOCX) [file pone.0349265.s002.docx]

S2 Table. Regression analysis between co-activation of upper trapezius muscles and cross-sectional neck-pain (n = 530)

|  | **Model 1** | | | **Model 2** | | | **Model 3** | | | **Model 4** | | |
| --- | --- | --- | --- | --- | --- | --- | --- | --- | --- | --- | --- | --- |
|  | **β** | **R2 (adj.)** | **p** | **β** | **R2 (adj.)** | **p** | **β** | **R2 (adj.)** | **p** | **β** | **R2 (adj.)** | **p** |
| 0 – 0.05% | **-0.176** | **0.029** | **< 0.001** | **-0.213** | **0.084** | **< 0.001** | **-0.220** | **0.079** | **< 0.001** | -0.084 | 0.018 |  |
| 0.05 – 2% | **-0.170** | **0.027** | **< 0.001** | **-0.177** | **0.072** | **< 0.001** | **-0.172** | **0.064** | **< 0.001** | 0.031 | 0.014 |  |
| 2 - 4% | -0.042 | 0.000 |  | -0.046 | 0.043 |  | -0.045 | 0.036 |  | 0.019 | 0.014 |  |
| 4 - 6% | 0.060 | 0.002 |  | 0.034 | 0.042 |  | 0.030 | 0.035 |  | 0.015 | 0.014 |  |
| 6 - 8% | **0.099** | **0.008** | **< 0.05** | 0.054 | 0.044 |  | 0.047 | 0.037 |  | 0.005 | 0.014 |  |
| 8 - 10% | **0.110** | **0.010** | **< 0.05** | 0.054 | 0.043 |  | 0.042 | 0.036 |  | 0.005 | 0.014 |  |
| 10 - 20% | 0.084 | 0.005 |  | 0.017 | 0.041 |  | 0.006 | 0.034 |  | -0.020 | 0.014 |  |
| 20 - 50% | 0.064 | 0.002 |  | -0.003 | 0.041 |  | -0.015 | 0.035 |  | -0.063 | 0.017 |  |
| 50 - 100% | 0.014 | -0.002 |  | -0.029 | 0.037 |  | -0.028 | 0.031 |  | -0.097 | 0.022 |  |
| ***Full range (0 - 100%)*** | -0.001 | -0.002 |  | -0.040 | 0.042 |  | -0.051 | 0.037 |  | 0.015 | 0.014 |  |

Model 1: Association between asymmetry in upper trapezius muscles activation and cross-sectional neck pain

Model 2: Model 1 adjusted for sex

Model 3: Model 1 adjusted for sex, height

Model 4: Model 1 adjusted for sex, height, smoking
