# Supplementary material for: Is asymmetric upper trapezius muscle activation during work associated with neck pain? A cross-sectional and longitudinal analysis
Source: PLoS One. 2026 Jun 12;21(6):e0349265. doi: 10.1371/journal.pone.0349265 (PMC13262944; doi:10.1371/journal.pone.0349265)
Supplement: S3 Table — Model 1: Association between asymmetry in upper trapezius muscles activation and longitudinal neck pain (unadjusted), Model 2: Model 1 adjusted for sex; Model 3: Model 1 adjusted for sex and height. (DOCX) [file pone.0349265.s003.docx]

S3 Table. Regression analysis between co-activation of upper trapezius muscles and longitudinal neck-pain (n = 256)

|  | **Model 1** | | | **Model 2** | | | **Model 3** | | |
| --- | --- | --- | --- | --- | --- | --- | --- | --- | --- |
|  | **β** | **R2 (adj.)** | **p** | **β** | **R2 (adj.)** | **p** | **β** | **R2 (adj.)** | **p** |
| 0 – 0.05% | -0.019 | -0.004 |  | -0.077 | 0.014 |  | -0.062 | 0.029 |  |
| 0.05 – 2% | 0.004 | -0.004 |  | -0.032 | 0.009 |  | -0.037 | 0.027 |  |
| 2 - 4% | 0.004 | -0.004 |  | -0.011 | 0.009 |  | -0.033 | 0.027 |  |
| 4 - 6% | 0.044 | -0.002 |  | 0.023 | 0.009 |  | 0.000 | 0.026 |  |
| 6 - 8% | 0.078 | 0.002 |  | 0.046 | 0.010 |  | 0.026 | 0.027 |  |
| 8 - 10% | 0.094 | 0.005 |  | 0.056 | 0.011 |  | 0.040 | 0.028 |  |
| 10 - 20% | 0.108 | 0.008 |  | 0.068 | 0.012 |  | 0.062 | 0.029 |  |
| 20 - 50% | **0.147** | **0.018** | **< 0.05** | 0.115 | 0.020 |  | 0.109 | 0.037 |  |
| 50 - 100% | 0.115 | 0.009 |  | 0.080 | 0.012 |  | 0.070 | 0.030 |  |
| ***Full range (0 - 100%)*** | 0.068 | 0.001 |  | 0.031 | 0.009 |  | 0.023 | 0.027 |  |

Model 1: Association between asymmetry in upper trapezius muscles activation and longitudinal neck pain (unadjusted)

Model 2: Model 1 adjusted for sex

Model 3: Model 1 adjusted for sex and height
