# Supplementary material for: Is asymmetric upper trapezius muscle activation during work associated with neck pain? A cross-sectional and longitudinal analysis
Source: PLoS One. 2026 Jun 12;21(6):e0349265. doi: 10.1371/journal.pone.0349265 (PMC13262944; doi:10.1371/journal.pone.0349265)
Supplement: Attachment — Submitted filename: Feedback.pdf [file pone.0349265.s005.pdf]

Does asymmetry in the activity of the upper trapezius muscles contribute to neck pain during work?

This manuscript investigates the association between asymmetric activation of the upper trapezius muscles during the workday and neck pain intensity using pooled electromyography (EMG) data from several Scandinavian research institutes. The study includes cross-sectional (n = 530) and longitudinal (n = 256) analyses and evaluates asymmetry across different muscle activation levels relative to maximal voluntary isometric contraction (MVIC).

The study addresses an interesting and underexplored question regarding bilateral muscle activation patterns in occupational settings. The dataset is relatively large and includes diverse occupational groups with full-day EMG recordings. Overall, the manuscript is well written and clearly structured.

Below are some suggestions that may help improve the manuscript.

Abstract
Your study is largely cross-sectional with limited predictive power. The following claim may therefore be too strong: *"asymmetry in the higher levels of muscle activity appears to be protective against neck pain."* Please consider using more cautious language, such as *"may be associated with."*

Given the limitations of the study, you may also consider adding a sentence such as:
"However, the explained variance of the models was small, and the results should therefore be interpreted with caution."

Introduction
Is the difference between **asymmetry** and **co-activation** clearly explained for the reader?

At the end of the introduction, please consider clarifying your hypothesis. For example:
"We hypothesized that asymmetry ..."

Methods
You combine datasets from seven institutes. While this is a strength, it may also introduce heterogeneity. Please consider providing more information on whether the measurement protocols were identical across sites and whether potential study-level differences were accounted for in the analyses.

You also converted different scales into VAS values. Since ordinal scales do not necessarily represent equal intervals, please consider adding a short justification for this decision.

Results
Statistical significance is reported, but the effect sizes are not interpreted. For example, the β

and R^2 values appear small. Although you acknowledge that the explained variances were minimal, this should also be more clearly highlighted in the Results section.

In addition, the Results could more clearly distinguish between cross-sectional and longitudinal findings. The longitudinal results appear weaker and could be emphasized as such.

Discussion

You propose that asymmetry during higher activity levels may be beneficial. While this is an interesting interpretation, it remains speculative. Please consider adding a clearer caution, for example: “These interpretations remain speculative and should be confirmed in future experimental studies.”

You correctly discuss the possibility of reverse causality. However, given its importance, it might be useful to introduce this limitation earlier in the discussion.

You report that smoking had a negative association with neck pain. This is a somewhat surprising finding and may warrant a more thorough discussion.

Although you acknowledge that the explained variances were minimal, this limitation could be discussed more explicitly.

Conclusion

The current conclusion “asymmetry in higher activity appears protective against neck pain”—may be too strong given the low explained variance, mixed results, and the design of the study. Please consider softening this statement.
