# Supplementary material for: Is asymmetric upper trapezius muscle activation during work associated with neck pain? A cross-sectional and longitudinal analysis
Source: PLoS One. 2026 Jun 12;21(6):e0349265. doi: 10.1371/journal.pone.0349265 (PMC13262944; doi:10.1371/journal.pone.0349265)
Supplement: Attachment — Submitted filename: Response to reviewers.docx [file pone.0349265.s006.docx]

PONE-D-25-62280

Does asymmetry in the activity of the upper trapezius muscles contribute to neck pain during work?

Thank you for submitting your manuscript to PLOS ONE. After careful consideration, we feel that it has merit but does not fully meet PLOS ONE’s publication criteria as it currently stands. Therefore, we invite you to submit a revised version of the manuscript that addresses the points raised during the review process.

**Please submit your revised manuscript by Apr 30 2026 11:59PM.**

***Please include the following items when submitting your revised manuscript:***

- A letter that responds to each point raised by the academic editor and reviewer(s). You should upload this letter as a separate file labeled 'Response to Reviewers'.
- A marked-up copy of your manuscript that highlights changes made to the original version. You should upload this as a separate file labeled 'Revised Manuscript with Track Changes'.
- An unmarked version of your revised paper without tracked changes. You should upload this as a separate file labeled 'Manuscript'.

Gianpiero Greco
Academic Editor
PLOS One

# Journal Requirements:

**When submitting your revision, we need you to address these additional requirements.
1. Please ensure that your manuscript meets PLOS ONE's style requirements, including those for file naming. The PLOS ONE style templates can be found at**
[**https://journals.plos.org/plosone/s/file?id=wjVg/PLOSOne_formatting_sample_main_body.pdf**](https://track.editorialmanager.com/CL0/https:%2F%2Fjournals.plos.org%2Fplosone%2Fs%2Ffile%3Fid=wjVg%2FPLOSOne_formatting_sample_main_body.pdf/1/010f019cf5157bed-df2a5fe8-d096-43a0-81d8-d2efa28ad9e4-000000/IDVrdViftgMN5MyhhHPQYaped-ARFl5UY0Rdpw5UFk8=254) **and**

[**https://journals.plos.org/plosone/s/file?id=ba62/PLOSOne_formatting_sample_title_authors_affiliations.pdf**](https://journals.plos.org/plosone/s/file?id=ba62/PLOSOne_formatting_sample_title_authors_affiliations.pdf)

**Revision:**	We have adapted the formatting to comply with the style requirements of PLOS ONE.

**2. When completing the data availability statement of the submission form, you indicated that you will make your data available on acceptance. We strongly recommend all authors decide on a data sharing plan before acceptance, as the process can be lengthy and hold up publication timelines. Please note that, though access restrictions are acceptable now, your entire data will need to be made freely accessible if your manuscript is accepted for publication. This policy applies to all data except where public deposition would breach compliance with the protocol approved by your research ethics board. If you are unable to adhere to our open data policy, please kindly revise your statement to explain your reasoning and we will seek the editor's input on an exemption. Please be assured that, once you have provided your new statement, the assessment of your exemption will not hold up the peer review process.**

**Revision:**	The data will be available via the Data file uploaded (S4_Dataset).

**3.** [**Please include captions for your Supporting Information files at the end of your manuscript, and update any in-text citations to match accordingly. Please see our Supporting Information guidelines for more information: http://journals.plos.org/plosone/s/supporting-information.**](http://track.editorialmanager.com/CL0/http:%2F%2Fjournals.plos.org%2Fplosone%2Fs%2Fsupporting-information./1/010f019cf5157bed-df2a5fe8-d096-43a0-81d8-d2efa28ad9e4-000000/ldNptZdpkG2nb33UG4NzN2-VGfhMQgSmDIp7q9qFvis=254)

Revision:	We included the captions after the reference list as follows:

**S1 Table. Regression analyses between individual factors and neck pain.**

**S2 Table.** **Regression analysis between co-activation of upper trapezius muscles and cross-sectional neck-pain (n = 530).** Model 1: Association between asymmetry in upper trapezius muscles activation and cross-sectional neck pain; Model 2: Model 1 adjusted for sex; Model 3: Model 1 adjusted for sex, height; Model 4: Model 1 adjusted for sex, height, smoking

**S3 Table. Regression analysis between co-activation of upper trapezius muscles and longitudinal neck-pain (n = 256).** Model 1: Association between asymmetry in upper trapezius muscles activation and longitudinal neck pain (unadjusted), Model 2: Model 1 adjusted for sex; Model 3: Model 1 adjusted for sex and height

**4. We are unable to open your Supporting Information file [S1_Dataset.sav]. Please kindly revise as necessary and re-upload.**

**Revision:**	We re-uploaded the datafile (S4_Dataset.sav) in SPSS format.

**5. We notice that your supplementary [tables] are included in the manuscript file. Please remove them and upload them with the file type 'Supporting Information'. Please ensure that each Supporting Information file has a legend listed in the manuscript after the references list.**

**Revision:**	We removed the supporting tables from the manuscript and uploaded them separately as supporting information.

6. If the reviewer comments include a recommendation to cite specific previously published works, please review and evaluate these publications to determine whether they are relevant and should be cited. There is no requirement to cite these works unless the editor has indicated otherwise.

Revision:	No revisions were made.

**7. Please review your reference list to ensure that it is complete and correct. If you have cited papers that have been retracted, please include the rationale for doing so in the manuscript text, or remove these references and replace them with relevant current references. Any changes to the reference list should be mentioned in the rebuttal letter that accompanies your revised manuscript. If you need to cite a retracted article, indicate the article’s retracted status in the References list and also include a citation and full reference for the retraction notice.**

**Revision:**	The reference list was checked for completeness and correctness. No revisions were made.

**Reviewer #1: the asymmetry in upper trapezius active as a source of neck pain is an interesting hypothesis**

General comments:

The paper is well written. The effort to combine and extract asymmetry information form a very large data set is commendable. However, this reviewer, like the authors, has a doubt on the conclusion "that asymmetry in the very low levels of muscle activity (muscular rest and static activities) may increase neck pain intensity". In several instances the authors, apparently, provide information relative to other studies or within their own results that asymmetry may not be the issue and that other factors may be masked, rendering the statistical analysis difficult to interpret since causality may not be as hypothesized. **Hence, asymmetry may be too strongly emphasized.**

**Reply:**	We have made changes to the manuscript in line with your comments and those of the second reviewer, including revising the conclusion. We therefore hope that by ‘softening’ the conclusion, our manuscript will appear more coherent.

As several of the authors have already provided ample support to the relationship between sustained low level of muscle activity and pain and the alleviation of pain by intermittent higher levels of muscle activity then it may be suspected that these phenomena are likely the major underlying mechanisms leading to pain and not so much asymmetry. It would be important to reflect mode adequately the title "Does asymmetry..." to attenuate the responsibility of asymmetry or to provide stronger evidence concerning "asymmetry"

**Reply:**	The authors softened the title to acknowledge that asymmetry is being explored rather than asserted as causal.

**Is asymmetric upper trapezius muscle activation during work associated with neck pain? A cross-sectional and longitudinal analysis.**

minor comments:

**L105-6	strange syntax "was anonymized accessed"**

***Revision:	“****The protocol of this study provides a comprehensive overview of the individual measurement protocols employed in the included studies, along with a detailed exposition of the methodology employed to address existing differences (38). The data contained in each individual study was collected between 01.11.2021 and 30.06.2022 in anonymized form.”*

**L212	"Participants had" suggestion: the average age of .......
in this study, is the description of population a real result or a part inherent to the methods??**

***Revision:***	We revised the sentence and moved it to the method section:

*“The average age, weight, height and BMI of the participants were 39.2 years	(SD 12.5 years), 75.4 kg (SD 15.1 kg), 173.7 cm (SD 9.6 cm), and 24.9 kg/m2	(SD 3.6) (see Table 2).”*

**L215	the sentence is missing something or " respectively" is not sin the correct place. Suggestion: On average participants rated neck pain intensity xx and YY in X-sec and in Long. studies, respectively**

***Revision:	“****On average participants rated neck pain intensity 1.7 (SD 2.3) and 1.9 (SD	2.0) in cross-sectional in longitudinal measures.”*

**L217	the average (is it an overall average over all participants**

***Revision:***	*“Across all participants, the average asymmetry in activation of both upper trapezius muscles during the working day was 51.6 % (SD 7.8%) and the average highest side activity was 7.3 % (SD 3.9 %).* “

**L219	in relation to "their respective" MVIC**

***Revision:	“****For the various levels of muscle activity in relation to their respective MVIC	the mean asymmetry during the working day varied from 45.1 % (SD 12.8 %)	to 60.6% (SD 18,8 %) (see Table 2 and Fig. 1).”*

**L217-20	considering the noise in signals (even after reduction process) and noise in the motor drive, are the differences a reliable indication of asymmetry (see general comments).**

***Revision:***	We added the following paragraph in the discussion (Strength and limitations):

*“During the process of quality control, the noise present in the signal of the activation of the upper trapezius muscles was systematically removed from all channels. Furthermore, both signals (left and right muscle activity) were measured in individual sub-studies using a single measuring device. Given that the noise level was typically less than 1% MVIC, the impact of an incorrect calculation of the noise level on the asymmetry values can be considered negligible. The repercussions of such an error would be most significant in areas exhibiting minimal muscle activation. However, given the tendency of asymmetry values to increase from areas of low muscle activation to those of high muscle activation, it can be assumed that the asymmetry values can be regarded as reliable.”*

**L263	"our study" /present study; and "was with values" (awkward form)**

***Revision:	“****The values of the neck pain intensity measured in our study were 1.7 (SD 2.3) and 1.9 (SD 2.0) on the VAS for cross-sectional and longitudinal measures, respectively. These values are lower compared to those of population-based studies in Denmark (49) or Norway (50).”*

**L264	same remark as before about syntax and use of respectively (something missing)**

***Revision:***	Please see revision before (L263).

**L267-70	This hypothesis should not be proposed since it has no basis of support. (IF").**

***Revision:***	The authors removed this hypothesis.

**L277	after manual task may add ref to strength support of argument**

***Revision:***	The authors added a reference.

**L277	"day spent in .. muscle activity" syntax please**

***Revision:	“****Since one can assume that most of the working day is spent with lower muscle activity levels, the average highest side activity is comprehensible.”*

**L280	"the study calculated" certainly not, but YOU did**

***Revision:***	*“On average, the total population was found to have a static level (10th percentile) and a peak level (90th percentile) of 1.5 %MVIC (SD 1.0 %MVIC) and 14 %MVIC (5 %MVIC), respectively.”*

**L282	"center" may not be appropriate word**

***Revision:***	We changed this word to the “average highest side activity” as called in the Methods.

*“The average highest side of muscle activity, which we determined, lies within this range.”*

**L284	since study included diverse populations this can be added to strengthen argument (as indicated by the authors latter on).**

***Revision:***	We added a sentence at the end of the paragraph “Limitations” that discussed the issues regarding the low magnitude of explained variances:

“*Despite the identification of several substantial associations, the corresponding values for the explained variances in neck pain intensity were found of a small magnitude. Consequently, the influence of a potential asymmetric activation in the upper trapezius muscle on neck pain intensity is estimated to be low. In contrast to laboratory studies, in which specific exposures can be tested, the physical, psychological, or social stresses present in the workplace are wide. The present study was not equipped to provide precise information regarding these exposures. Nevertheless, this wide range of potential risk factors may have contributed to a lower level of variance. While this may be considered a disadvantage, it could also imply that the significant associations identified can be generalized.”*

**L291	"Paradoxical" it may not be a paradox since data suggest that higher activity as opposed to static low level has a beneficial effect on (it has been suggested that disruption of monotonic activity by higher activity (change in recruitment) is beneficial.**

***Revision:***	The word “paradoxical” was changed to “differential”:

“*Our cross-sectional analyses revealed a differential relationship between asymmetry and neck pain intensity across different muscle activation levels.”*

**L298	this send back to comment about asymmetry (may be sustained low level not appearing in asymmetry computation)**

***Revision:***	We revised the last part of the corresponding paragraph:

“*Asymmetric patterns during rest periods may indicate underlying neuromuscular dysfunction, compensation strategies, or inadequate recovery, potentially contributing to tissue stress and pain development. However, it should be noted that the observed association may not be attributable to asymmetry itself, but rather to sustained low-level muscle activity or insufficient muscular rest, both of which have previously been linked to neck pain (13, 17). The asymmetry measure at these very low activation levels may therefore partly capture these established risk factors rather than reflecting an independent effect of bilateral imbalance.”*

**L297-302	see comments about asymmetry (as presented it may be misleading/biased)**

***Revision:***	Please see revision before (L298).

**L310	confounding factors. Indeed agreed so see comment about prudence concerning asymmetric assertion. The 2 paragraphs may come late since they contribute to cast some doubt and weaken the proposed conclusion.**

***Revision:	We added the following sentence:***

“*These interpretations remain speculative and should be confirmed in future experimental studies.”*

**L 377	Indeed the VAS transformation may have biased pain intensity measure**

***Revision:***	The authors added the sentence:

“*This transformation may have biased the analysis of the association between the asymmetry of muscle activity and pain intensity measures.”*

**L402	the authors recommend caution but not strongly enough since conclusion does not**

***Revision:	We deleted the sentence “***Given the multifactorial nature of neck pain, the results and conclusions of this study must be viewed with caution.” in the paragraph of “Strengths and limitations and changed the conclusion of the manuscript as follows:

*“By integrating data sets from multiple previous studies, this study examined the association between upper trapezius muscle activity and neck pain intensity, with a focus on the asymmetric activation of these muscles. In contrast to the findings of preceding studies, a novel approach that has not yet been considered was investigated. The results of this study point towards, that asymmetry in the very low levels of muscle activity (muscular rest and static activities) may increase neck pain intensity, while asymmetry in the higher levels of muscle activity appears to be protective against neck pain. In general, the variance explained of the models was small, and the results should therefore be interpreted with caution. The findings suggest that work conditions facilitating simultaneous relaxation during breaks and balanced activation of both muscles during static activities may be relevant for the prevention of neck pain, though further research is needed to establish causality and, if this is the case, to determine what constitutes a 'normal' or 'safe' level of asymmetrical work.*“

**Reviewer #2: Does asymmetry in the activity of the upper trapezius muscles contribute to neck pain during work?**

This manuscript investigates the association between asymmetric activation of the upper trapezius muscles during the workday and neck pain intensity using pooled electromyography (EMG) data from several Scandinavian research institutes. The study includes cross-sectional (n = 530) and longitudinal (n = 256) analyses and evaluates asymmetry across different muscle activation levels relative to maximal voluntary isometric contraction (MVIC).

The study addresses an interesting and underexplored question regarding bilateral muscle activation patterns in occupational settings. The dataset is relatively large and includes diverse occupational groups with full-day EMG recordings. Overall, the manuscript is well written and clearly structured.

Below are some suggestions that may help improve the manuscript.

Abstract
**Your study is largely cross-sectional with limited predictive power. The following claim may therefore be too strong: “asymmetry in the higher levels of muscle activity appears to be protective against neck pain.” Please consider using more cautious language, such as “may be associated with.”**

***Revision:***	We revised / adapted the language of in the conclusion:

“***Conclusion*** *While asymmetry in the very low levels of muscle activity may be associated with higher neck pain intensity, asymmetry in the higher levels of muscle activity was negatively associated with neck pain intensity. However, the explained variance of the models was small, and the results should therefore be interpreted with caution. The findings suggest that work conditions facilitating simultaneous relaxation during breaks and balanced activation of both muscles during static activities may be relevant for neck pain prevention, though further research is needed to establish causality.”*

**Given the limitations of the study, you may also consider adding a sentence such as:
“However, the explained variance of the models was small, and the results should therefore be interpreted with caution.”**

***Revision:	“***We revised the conclusion of the abstract. And this sentence was included in the conclusion of the abstract. Due to maximal word count (max. 300 words), we had to revise parts of the abstract:

***Objectives*** *Previous studies have linked activity in the upper trapezius muscle with neck pain. However, no studies have examined whether asymmetric activation of these muscles during the working day is associated with neck pain. This study aimed to investigate this relationship.*

***Methods*** *Seven research institutes provided data on bilateral upper trapezius muscle activity on one working day, along with corresponding questionnaire data on cross-sectional (n = 530) and longitudinal (n = 256) neck pain intensity. The asymmetry, defined as the activity difference between the two upper trapezius muscles, was calculated as an average across the entire workday and within various intensity levels in relation to maximum voluntary isometric contraction (MVIC). Unadjusted and adjusted linear regression analyses were executed to examine the association between asymmetric muscle activation and neck pain intensity.*

***Results*** *In cross-sectional analyses, asymmetry in the levels 0 - 0.05 and 0.05 – 2 %MVIC was significantly positively associated with neck pain intensity in both unadjusted and adjusted analyses. Asymmetry in the levels of 4 - 6, 6 - 8 and 8 – 10 %MVIC was significantly negatively associated with neck pain in unadjusted analyses. In longitudinal analyses, significant positive associations were found for asymmetry in level 0 - 0.05 %MVIC and negative associations for asymmetry in levels > 20 %MVIC.*

***Conclusion*** *While asymmetry in the very low levels of muscle activity may be associated with higher neck pain intensity, asymmetry in the higher levels of muscle activity was negatively associated with neck pain intensity. However, the explained variance of the models was small, and the results should therefore be interpreted with caution. The findings suggest that work conditions facilitating simultaneous relaxation during breaks and balanced activation of both muscles during static activities may be relevant for neck pain prevention, though further research is needed to establish causality.”*

Introduction

**Is the difference between asymmetry and co-activation clearly explained for the reader?**

***Revision:***	We added the following sentence in the introduction:

“*Co-activation refers to the simultaneous contraction of agonist–antagonist muscles, where muscles on both sides of the same joint are active together. Asymmetry, by contrast, captures the degree to which one side's activation (right vs left) differs from the other at a given moment.”*

**At the end of the introduction, please consider clarifying your hypothesis. For example: “We hypothesized that asymmetry …”**

***Revision:***	We added the following sentence at the end of the introduction:

“*We hypothesized that an asymmetry in activation of the upper trapezius muscle is associated with neck pain intensity.”*

Methods

You combine datasets from seven institutes. While this is a strength, it may also introduce heterogeneity. Please consider providing more information on whether the measurement protocols were identical across sites and whether potential study-level differences were accounted for in the analyses.

***Revision:***	We included the following sentence in the methods section:

“*The protocol of this study provides a comprehensive overview of the individual measurement protocols employed in the included studies, along with a detailed exposition of the methodology employed to address existing differences (38).”*

**You also converted different scales into VAS values. Since ordinal scales do not necessarily represent equal intervals, please consider adding a short justification for this decision.**

***Revision:***	We added the following sentence to the paragraph:

“*The VAS was selected as the primary scale for the pooled dataset with a view to averting any further deterioration in the quality of the pain measurements. The allocation of individual categorical responses to corresponding numerical values on the VAS was determined by consent among all co-authors based on a "best fit".”*

Results

**(1) Statistical significance is reported, but the effect sizes are not interpreted. For example, the β and R² values appear small. (2) Although you acknowledge that the explained variances were minimal, this should also be more clearly highlighted in the Results section.**

***Revision:	1.*** We added the following sentences for the association between the side-independent, respectively the side dependent asymmetry and neck pain intensity:

“*The significant associations that were identified were found to be of a small magnitude (β < 0.181), as was the variance in neck pain intensity that was explained (R² < 0.088).”*

*“The significant associations that were identified were found to be of a small magnitude (β < 0.103) and explained a low variance in neck pain intensity (R² < 0.011).”*

***2.*** *We added the following sentences in the Strength and limitations section in the Discussion:*

“*Nevertheless, this wide range of potential risk factors may have contributed to a lower level of variance. While this may be considered a disadvantage, it could also imply that the significant associations identified can be generalized.”*

**In addition, the Results could more clearly distinguish between cross-sectional and longitudinal findings. The longitudinal results appear weaker and could be emphasized as such.**

***Revision:***	We added the following sentence under the heading “Results - Longitudinal	analyses of asymmetry of muscle activation and neck pain intensity”:

“*Compared to the cross-sectional analyses, the longitudinal analyses yielded fewer and weaker significant associations overall.”*

# Discussion

**You propose that asymmetry during higher activity levels may be beneficial. While this is an interesting interpretation, it remains speculative. Please consider adding a clearer caution, for example: “These interpretations remain speculative and should be confirmed in future experimental studies.”**

***Revision:***	We insert this sentence in the discussion under the heading “Cross-	sectional association between asymmetric muscle activation and neck	pain intensity”

***“****These interpretations remain speculative and should be confirmed in future experimental studies.”*

**You correctly discuss the possibility of reverse causality. However, given its importance, it might be useful to introduce this limitation earlier in the discussion.**

***Revision:	“***We moved the relevant paragraph to the section Discussion (Cross-sectional association between asymmetric muscle activation and neck pain intensity).”

**You report that smoking had a negative association with neck pain. This is a somewhat surprising finding and may warrant a more thorough discussion.**

***Revision:	We added the following sentence:***

“*One possible explanation for the negative associations between smoking and neck pain intensity might be the opportunity to rest during smoking breaks. It is also conceivable that the pain is alleviated by increased social interaction during these breaks (4). Due to the small number of smokers (n = 128) in our sample, this association was not investigated further.”*

**Although you acknowledge that the explained variances were minimal, this limitation could be discussed more explicitly.**

***Revision:***	We revised the paragraph as follows:

“*Despite the identification of several substantial associations, the corresponding values for the explained variances in neck pain intensity were found of a small magnitude. Consequently, the influence of a potential asymmetric activation in the upper trapezius muscle on neck pain intensity is estimated to be low. In contrast to laboratory studies, in which specific exposures can be tested, the physical, psychological, or social stresses present in the workplace are wide. The present study was not equipped to provide precise information regarding these exposures. Nevertheless, this wide range of potential risk factors may have contributed to a lower level of variance. While this may be considered a disadvantage, it could also imply that the significant associations identified can be generalized.”*

# Conclusion

**The current conclusion “asymmetry in higher activity appears protective against neck pain”—may be too strong given the low explained variance, mixed results, and the design of the study. Please consider softening this statement.**

***Revision:***	We revised the conclusion as follows:

*By integrating data sets from multiple previous studies, this study examined the association between upper trapezius muscle activity and neck pain intensity, with a focus on the asymmetric activation of these muscles. In contrast to the findings of preceding studies, a novel approach that has not yet been considered was investigated. The results of this study point towards, that asymmetry in the very low levels of muscle activity (muscular rest and static activities) may increase neck pain intensity, while asymmetry in the higher levels of muscle activity appears to be protective against neck pain. In general, the variance explained of the models was small, and the results should therefore be interpreted with caution. The findings suggest that work conditions facilitating simultaneous relaxation during breaks and balanced activation of both muscles during static activities may be relevant for the prevention of neck pain, though further research is needed to establish causality and, if this is the case, to determine what constitutes a 'normal' or 'safe' level of asymmetrical work.*
