# Supplementary material for: Is asymmetric upper trapezius muscle activation during work associated with neck pain? A cross-sectional and longitudinal analysis
Source: PLoS One. 2026 Jun 12;21(6):e0349265. doi: 10.1371/journal.pone.0349265 (PMC13262944; doi:10.1371/journal.pone.0349265)
Supplement: Peer review history

# Peer review: Decision Letter 0

**DOI:** 10.1371/journal.pone.0349265.r001

**Authors:** Gianpiero Greco

16 Mar 2026

PONE-D-25-62280

Does asymmetry in the activity of the upper trapezius muscles contribute to neck pain during work?

PLOS One

Dear Dr. Koch,

Thank you for submitting your manuscript to PLOS ONE. After careful consideration, we feel that it has merit but does not fully meet PLOS ONE’s publication criteria as it currently stands. Therefore, we invite you to submit a revised version of the manuscript that addresses the points raised during the review process.

Please submit your revised manuscript by Apr 30 2026 11:59PM. If you will need more time than this to complete your revisions, please reply to this message or contact the journal office at plosone@plos.org. When you're ready to submit your revision, log on to https://www.editorialmanager.com/pone/ and select the 'Submissions Needing Revision' folder to locate your manuscript file.

Please include the following items when submitting your revised manuscript:

- A letter that responds to each point raised by the academic editor and reviewer(s). You should upload this letter as a separate file labeled 'Response to Reviewers'.
- A marked-up copy of your manuscript that highlights changes made to the original version. You should upload this as a separate file labeled 'Revised Manuscript with Track Changes'.
- An unmarked version of your revised paper without tracked changes. You should upload this as a separate file labeled 'Manuscript'.

-->If you would like to make changes to your financial disclosure, please include your updated statement in your cover letter. Guidelines for resubmitting your figure files are available below the reviewer comments at the end of this letter.

If applicable, we recommend that you deposit your laboratory protocols in protocols.io to enhance the reproducibility of your results. Protocols.io assigns your protocol its own identifier (DOI) so that it can be cited independently in the future. For instructions see: https://journals.plos.org/plosone/s/submission-guidelines#loc-laboratory-protocols. Additionally, PLOS ONE offers an option for publishing peer-reviewed Lab Protocol articles, which describe protocols hosted on protocols.io. Read more information on sharing protocols at https://plos.org/protocols?utm_medium=editorial-email&utm_source=authorletters&utm_campaign=protocols.

We look forward to receiving your revised manuscript.

Kind regards,

Gianpiero Greco

Academic Editor

PLOS One

Journal Requirements:

When submitting your revision, we need you to address these additional requirements.

1. Please ensure that your manuscript meets PLOS ONE's style requirements, including those for file naming. The PLOS ONE style templates can be found at

https://journals.plos.org/plosone/s/file?id=wjVg/PLOSOne_formatting_sample_main_body.pdf and https://journals.plos.org/plosone/s/file?id=ba62/PLOSOne_formatting_sample_title_authors_affiliations.pdf

2. When completing the data availability statement of the submission form, you indicated that you will make your data available on acceptance. We strongly recommend all authors decide on a data sharing plan before acceptance, as the process can be lengthy and hold up publication timelines. Please note that, though access restrictions are acceptable now, your entire data will need to be made freely accessible if your manuscript is accepted for publication. This policy applies to all data except where public deposition would breach compliance with the protocol approved by your research ethics board. If you are unable to adhere to our open data policy, please kindly revise your statement to explain your reasoning and we will seek the editor's input on an exemption. Please be assured that, once you have provided your new statement, the assessment of your exemption will not hold up the peer review process.

3. Please include captions for your Supporting Information files at the end of your manuscript, and update any in-text citations to match accordingly. Please see our Supporting Information guidelines for more information: http://journals.plos.org/plosone/s/supporting-information.

4. We are unable to open your Supporting Information file [S1_Dataset.sav]. Please kindly revise as necessary and re-upload.

5. We notice that your supplementary [tables] are included in the manuscript file. Please remove them and upload them with the file type 'Supporting Information'. Please ensure that each Supporting Information file has a legend listed in the manuscript after the references list.

6. If the reviewer comments include a recommendation to cite specific previously published works, please review and evaluate these publications to determine whether they are relevant and should be cited. There is no requirement to cite these works unless the editor has indicated otherwise.

7. Please review your reference list to ensure that it is complete and correct. If you have cited papers that have been retracted, please include the rationale for doing so in the manuscript text, or remove these references and replace them with relevant current references. Any changes to the reference list should be mentioned in the rebuttal letter that accompanies your revised manuscript. If you need to cite a retracted article, indicate the article’s retracted status in the References list and also include a citation and full reference for the retraction notice.

[Note: HTML markup is below. Please do not edit.]

Reviewers' comments:

Reviewer's Responses to Questions

**Comments to the Author**

1. Is the manuscript technically sound, and do the data support the conclusions?

The manuscript must describe a technically sound piece of scientific research with data that supports the conclusions. Experiments must have been conducted rigorously, with appropriate controls, replication, and sample sizes. The conclusions must be drawn appropriately based on the data presented.

Reviewer #1: Partly

Reviewer #2: Yes

**********

2. Has the statistical analysis been performed appropriately and rigorously?

Reviewer #1: Yes

Reviewer #2: Yes

**********

3. Have the authors made all data underlying the findings in their manuscript fully available?

The PLOS Data policy requires authors to make all data underlying the findings described in their manuscript fully available without restriction, with rare exception (please refer to the Data Availability Statement in the manuscript PDF file). The data should be provided as part of the manuscript or its supporting information, or deposited to a public repository. For example, in addition to summary statistics, the data points behind means, medians and variance measures should be available. If there are restrictions on publicly sharing data—e.g. participant privacy or use of data from a third party—those must be specified.

Reviewer #1: Yes

Reviewer #2: No

**********

4. Is the manuscript presented in an intelligible fashion and written in standard English?

PLOS ONE does not copyedit accepted manuscripts, so the language in submitted articles must be clear, correct, and unambiguous. Any typographical or grammatical errors should be corrected at revision, so please note any specific errors here.

Reviewer #1: Yes

Reviewer #2: Yes

**********

5. Review Comments to the Author

Please use the space provided to explain your answers to the questions above. You may also include additional comments for the author, including concerns about dual publication, research ethics, or publication ethics. (Please upload your review as an attachment if it exceeds 20,000 characters)

Reviewer #1: the asymmetry in upper trapezius active as a source of neck pain is an interesting hypothesis

General comments:

the paper is well written. the effort to combine and extract asymmetry information form a very large data set is commendable. However, This reviewer, like the authors, has a doubt on the conclusion "that

asymmetry in the very low levels of muscle activity (muscular rest and static activities) may.increase neck pain intensity". In several instances the authors , apparently, provide information relative to other studies or within their own results that asymmetry may not be the issue and that other factors may be masked, rendering the statistical analysis difficult to interpret since causality may not be as hypothesized. Hence, asymmetry may be too strongly emphasized.

As several of the authors have already provided ample support to the relationship between sustained low level of muscle activity and pain and the alleviation of pain by intermittent higher levels of muscle activity then it may be suspected that these phenomena are likely the major underlying mechanisms leading to pain and not so much asymmetry. It would be important to reflect mode adequately the title "Does asymmetry..." to attenuate the responsibility of asymmetry or to provide stronger evidence concerning "asymmetry"

minor comments:

L105-6 strange syntax "was anonymized accessed"

L212 "Participants had" suggestion: the average age of .......

in this study, is the description of population a real results or a part inherent to the methods??

L215 the sentence is missing something or " respectively" is not sin the correct place. Suggestion : On average participants rated neck pain intensity xx and YY in X-sec and in Long. studies, respectively

L217. the average (is it an overall average over all participants

L219 in relation to "their respective" MVIC

L217-20 considering the noise in signals (even after reduction process) and noise in the motor drive, are the differences a reliable indication of asymmetry (see general comments)

l263 "our"study" /present study; and "was with values" ( awkward form)

L264 same remark as before about syntax and use of respectively (something missing)

L267-70. this hypothesis should not be proposed since it has no basis of support. (IF").

L277 after manual task may add ref to strength support of argument

L277 "day spent in .. muscle activity" syntax please

L280 "the study calculated" certainly not, but YOU did

L282 "center" may not be appropriate word

L284 since study included diverse populations this can be added to strengthen argument (as indicated by the authors latter on)

L291 "Paradoxical" it may not be a paradox since data suggest that higher activity as opposed to static low level has a beneficial effect on(it has been suggested that disruption of monotonic activity by higher activity ( change in recruitment) is beneficial.

L298 this send back to comment about asymmetry (may be sustained low level not appearing in asymmetry computation)

L297-302 see comments about asymmetry ( as presented it may be misleading/biased)

L310. confounding factors. _indeed agreed so see comment about prudence concerning asymmetric assertion. the 2 paragraphs may come late since they contribute to cast some doubt and weaken the proposed conclusion

L 377. indeed the VAS transformation may have biased pain intensity measure

L402 the authors recommend caution but not strongly enough since conclusion does not

Reviewer #2: Does asymmetry in the activity of the upper trapezius muscles contribute to neck pain during work?

This manuscript investigates the association between asymmetric activation of the upper trapezius muscles during the workday and neck pain intensity using pooled electromyography (EMG) data from several Scandinavian research institutes. The study includes cross-sectional (n = 530) and longitudinal (n = 256) analyses and evaluates asymmetry across different muscle activation levels relative to maximal voluntary isometric contraction (MVIC).

The study addresses an interesting and underexplored question regarding bilateral muscle activation patterns in occupational settings. The dataset is relatively large and includes diverse occupational groups with full-day EMG recordings. Overall, the manuscript is well written and clearly structured.

Below are some suggestions that may help improve the manuscript.

Abstract

Your study is largely cross-sectional with limited predictive power. The following claim may therefore be too strong: “asymmetry in the higher levels of muscle activity appears to be protective against neck pain.” Please consider using more cautious language, such as “may be associated with.”

Given the limitations of the study, you may also consider adding a sentence such as:

“However, the explained variance of the models was small, and the results should therefore be interpreted with caution.”

Introduction

Is the difference between asymmetry and co-activation clearly explained for the reader?

At the end of the introduction, please consider clarifying your hypothesis. For example:

“We hypothesized that asymmetry …”

Methods You combine datasets from seven institutes. While this is a strength, it may also introduce heterogeneity. Please consider providing more information on whether the measurement protocols were identical across sites and whether potential study-level differences were accounted for in the analyses.

You also converted different scales into VAS values. Since ordinal scales do not necessarily represent equal intervals, please consider adding a short justification for this decision.

Results Statistical significance is reported, but the effect sizes are not interpreted. For example, the β and R² values appear small. Although you acknowledge that the explained variances were minimal, this should also be more clearly highlighted in the Results section.

In addition, the Results could more clearly distinguish between cross-sectional and longitudinal findings. The longitudinal results appear weaker and could be emphasized as such.

Discussion You propose that asymmetry during higher activity levels may be beneficial. While this is an interesting interpretation, it remains speculative. Please consider adding a clearer caution, for example: “These interpretations remain speculative and should be confirmed in future experimental studies.”

You correctly discuss the possibility of reverse causality. However, given its importance, it might be useful to introduce this limitation earlier in the discussion.

You report that smoking had a negative association with neck pain. This is a somewhat surprising finding and may warrant a more thorough discussion.

Although you acknowledge that the explained variances were minimal, this limitation could be discussed more explicitly.

Conclusion The current conclusion “asymmetry in higher activity appears protective against neck pain”—may be too strong given the low explained variance, mixed results, and the design of the study. Please consider softening this statement.

**********

6. PLOS authors have the option to publish the peer review history of their article (what does this mean?). If published, this will include your full peer review and any attached files.

If you choose “no”, your identity will remain anonymous but your review may still be made public.

**Do you want your identity to be public for this peer review?**   For information about this choice, including consent withdrawal, please see our Privacy Policy.

Reviewer #1: No

Reviewer #2: No

**********

[NOTE: If reviewer comments were submitted as an attachment file, they will be attached to this email and accessible via the submission site. Please log into your account, locate the manuscript record, and check for the action link "View Attachments". If this link does not appear, there are no attachment files.]

To ensure your figures meet our technical requirements, please review our figure guidelines: https://journals.plos.org/plosone/s/figures

You may also use PLOS’s free figure tool, NAAS, to help you prepare publication quality figures: https://journals.plos.org/plosone/s/figures#loc-tools-for-figure-preparation.

NAAS will assess whether your figures meet our technical requirements by comparing each figure against our figure specifications.

Attachment Submitted filename: Feedback.pdf

# Author comment: Author response to Decision Letter 1

**DOI:** 10.1371/journal.pone.0349265.r002

20 Apr 2026

The responses to the reviewers comments are available in the "response to reviewers" file.

Attachment Submitted filename: Response to reviewers.docx

# Editor report: Decision Letter 1

**DOI:** 10.1371/journal.pone.0349265.r003

**Authors:** Gianpiero Greco

27 Apr 2026

Is asymmetric upper trapezius muscle activation during work associated with neck pain? A cross-sectional and longitudinal analysis.

PONE-D-25-62280R1

Dear Dr. Koch,

We’re pleased to inform you that your manuscript has been judged scientifically suitable for publication and will be formally accepted for publication once it meets all outstanding technical requirements.

Within one week, you’ll receive an e-mail detailing the required amendments. When these have been addressed, you’ll receive a formal acceptance letter and your manuscript will be scheduled for publication.

An invoice will be generated when your article is formally accepted. Please note, if your institution has a publishing partnership with PLOS and your article meets the relevant criteria, all or part of your publication costs will be covered. Please make sure your user information is up-to-date by logging into Editorial Manager at Editorial Manager® and clicking the ‘Update My Information' link at the top of the page. For questions related to billing, please contact billing support.

If your institution or institutions have a press office, please notify them about your upcoming paper to help maximize its impact. If they’ll be preparing press materials, please inform our press team as soon as possible -- no later than 48 hours after receiving the formal acceptance. Your manuscript will remain under strict press embargo until 2 pm Eastern Time on the date of publication. For more information, please contact onepress@plos.org.

Kind regards,

Gianpiero Greco

Academic Editor

PLOS One

Additional Editor Comments (optional):

Reviewers' comments:

# Editor report: Acceptance letter

**DOI:** 10.1371/journal.pone.0349265.r004

**Authors:** Gianpiero Greco

PONE-D-25-62280R1

PLOS One

Dear Dr. Koch,

I'm pleased to inform you that your manuscript has been deemed suitable for publication in PLOS One. Congratulations! Your manuscript is now being handed over to our production team.

At this stage, our production department will prepare your paper for publication. This includes ensuring the following:

* All references, tables, and figures are properly cited

* All relevant supporting information is included in the manuscript submission,

* There are no issues that prevent the paper from being properly typeset

You will receive further instructions from the production team, including instructions on how to review your proof when it is ready. Please keep in mind that we are working through a large volume of accepted articles, so please give us a few days to review your paper and let you know the next and final steps.

Lastly, if your institution or institutions have a press office, please let them know about your upcoming paper now to help maximize its impact. If they'll be preparing press materials, please inform our press team within the next 48 hours. Your manuscript will remain under strict press embargo until 2 pm Eastern Time on the date of publication. For more information, please contact onepress@plos.org.

You will receive an invoice from PLOS for your publication fee after your manuscript has reached the completed accept phase. If you receive an email requesting payment before acceptance or for any other service, this may be a phishing scheme. Learn how to identify phishing emails and protect your accounts at https://explore.plos.org/phishing.

If we can help with anything else, please email us at customercare@plos.org.

Thank you for submitting your work to PLOS ONE and supporting open access.

Kind regards,

PLOS ONE Editorial Office Staff

on behalf of

Dr. Gianpiero Greco

Academic Editor

PLOS One
